# "The Hospital" Nursing Mirror

**Published:** 1900-11-17

**Authors:** 


"The Hospital" Nursing Mirror.

Being the Nursing Section of "The Hospital."

[Contributions for this Section of "The Hospital" should be addressed to the Editor, "The Hospital" Nursing Mirror, 28 & 29 Southampton Street, Strand, London, W.C.]

### Notes on News from the Nursing World.

### THE QUEEN AND MAFEKING NURSES.

The Queen, who never loses an opportunity of showing her sympathy with all who are engaged in nursing, and especially with those who pursue this work under special difficulties or in face of peril, received on Tuesday, at Windsor Castle, Mother Superior Teresa and Sister Evangeline, who assisted in nursing the sick and wounded soldiers during the famous siege of Mafeking. The sisters, who were habited in the black dresses and white hoods and collars of the community to which they belong, were met by a Royal carriage, and on their arrival, about 1.30, conveyed to the Palace. Having partaken of luncheon, they were presented by the Hon. Mrs. Mallet to the Queen, who manifested the deepest interest in the work in which they were engaged for upwards of seven months. Princess Henry of Battenberg was present during the interesting proceedings. Both the Mother Superior and Sister Evangeline are returning in a few days to Mafeking, and they will carry with them to South Africa pleasing recollections of the gracious and kindly reception given them by the Sovereign.

### HOUSEMAIDS AS "NURSES" IN WORKHOUSE INFIRMARIES.

The reports of the inspectors under the Local Government Board on nursing in workhouses, published in our columns to-day, will be read with mixed feelings. Mr. Baldwyn Fleming reports that "gradual improvement may be noticed." Mr. Dansey shares his opinion, and expresses his satisfaction that guardians are turning their attention to the nursing of the outside poor. Mr. Dawson speaks of the nursing staff, "at any rate in the larger workhouses, being slowly strengthened; and Mr. Bircham, who supplies very scanty information, states that in the Principality matters are slowly improving in the larger establishments, "though, perhaps, not to the extent to be found in most English districts." On the other hand, Mr. Bagenal affirms that the difficulty in obtaining and retaining the services of nurses continues to increase. Mr. Wetherhed reports friction between masters and matrons, on the one part, and nurses of the infirmaries, on the other, and comments on the mischievous practice of putting young probationers on night duty by themselves. Lastly, Mr. Kennedy, in his most interesting report, refers to the deplorable results of appointing housemaids as nurses. This, he says, has been done in Yorkshire in consequence of qualified nurses naturally refusing to respond to an offer of £20 a year. He mentions an instance where, as the doctor declared, the "nurse" had never seen a confinement case before, and "had no idea what to do:" another, where the "nurse" being told to take a temperature in the course of the evening, put the clinical thermometer, which the doctor had left with her, into the chamber utensil; and a third case where, in endeavouring to pass a catheter, she caused the death of the patient. We trust that Mr. Walter Long, the new President of the Local Government Board, will take an early opportunity of giving his close attention to the reports of the inspectors, with the view of, at all events, preventing such a scandalous state of things as Mr. Kennedy vouches for.

### NURSING ON THE "BRITANNIC."

The *Britannic* had six nursing sisters on board on her latest voyage from the Cape, namely Sister-Superintendent McCall Anderson, and Sisters Monson, Shore, Rogers, Tucker, and Pryde. There were also four sisters who have been invalided home. The men were convalescent from enteric and rheumatism, and there were a few slight surgical cases. No illness developed on board. The "hospital" consisted of several cabins, each containing four bunks: these were allotted to the patients requiring the greatest care. Those who were further on the road towards recovery slept in swing cots on the lower troop deck, and those who were comparatively well in the ordinary hammocks. The sisters on duty took turns with the medical and surgical nursing. The food, we are informed, was exceedingly good, and every patient was on stimulant of some sort. One sergeant and four orderlies were on duty. After a fair voyage the patients were drafted to the various military hospitals.

### HOW THEY WERE TAKEN IN.

The following incident appeared on the night of the occupation of Bloemfontein. The rain, coming down in torrents, had driven all to shelter, of one kind or another, all but two officers, who having no blankets or other means of protection from the unkind heavens, determined to find a lodging by hook or by crook. "That's a fine big house—we'll go there," said No. 1. "Do you know who lives there?" mildly suggested No. 2. "Not I, but let's go and see." So, arriving at the door—the house looking rather deserted—they gave a resounding knock, and yet another; far away in the distance they could hear movements, and after a time a nurse in uniform, holding a candle, appeared. Feeling somewhat abashed they told their story, and asked if they could be taken in, conscious that their dirty and bedraggled appearance—they had tramped many miles—rather discounted what they said. After a somewhat doubtful survey of the would-be lodgers, the nurse said she would "go and see," by her manner suggesting they should remain where they were. However, this did not fall in with the feelings of No. 1, so he gently asked, "while she was going to see if they might stand inside out of the rain," to which the nurse had to assent. After a little further delay footsteps were heard, and a lady, apparently the matron, appeared. "Do you know where you are?" she asked, after the first preliminaries. Looking round the large clean hall, and then at the nursing uniform worn by the two, the officer answered: "I suppose this is a hospital?" "No," said the matron, "it is a lunatic asylum, and this is the wing for female inmates." Deprecating their suitability for admittance, they were very thankful when she invited them to come in for the night and said that she would find beds for them somehow. The novelty of tables, chairs, table linen, flowers, and the ordinary refinements of life, after weeks on the veldt, were delightful to them, but after a most excellent meal they found themselves, without reflection, sitting down quite naturally on the floor. They went to sleep to an accompaniment of groans and shrieks from *bonâ fide* lunatics.

### A UNIQUE CHRISTMAS PRESENT.

The box which reached a certain company of Tommies at Christmas last year might well have been labelled "Good Intentions." "One soldier," writes a nurse in South Africa, "described to me the share he had with two others in a small plum-pudding big enough for one. 'And what had you?' I said to a trooper. 'Oh, I had half a box of sardines to share with a chum, one cigarette, and a tin of insect powder; it was kindly intended'—with a wry face, half a smile, he added—'but inadequate; half a pailful of insect powder was what we wanted just there!'"

### THE PAY OF NURSES IN IRELAND.

With reference to our remarks about the pay of Irish nurses, we are not surprised to learn, from a correspondent, that the point at issue is no longer the pay but the remedy. In view of this fact it may be interesting to cite the scale of salaries of nurses attached to some of the well-known institutions. At the Adelaide Hospital (Protestant) probationers, who pay a fee of £5, receive for the first year £10, second £12, third £15, with indoor and outdoor uniform. The trained nurses receive £20 the first year, £23 the second, and £26 the third and subsequent years. The divisional nurses receive £30 a year, with an annual rise of £2 10s. till £40 is reached. At the Mater Misericordiæ (Roman Catholic) the fee is £20. No salary is paid the first year, £8 the second, £12 the third, £16 the fourth. A sister receives £20, but there is no further rise. At Dr. Steevens' Hospital (General) at present the payment the second year is £8, third £12, fourth £16, and fifth £20, increasing £2 each year to £40. The sisters in the wards also receive £40, and 2s. 6d. a week bonus on each case for private nurses. At Sir Patrick Dun's Hospital the probationers receive £10 the first year, to be increased £2 every year till £30 is reached, which is the limit. Staff nurs s have from £30 to £35. Indoor uniform provided, no outdoor. At the Hardwick, Whitworth, and Richmond Hospitals the probationers receive £10 the first year, increasing to £30, which is the limit. If the Irish nurse appeals for an increase of salary, she is invariably told that when the Irish people are willing to pay more than £1 1s. for general cases and £1 11s. 6d. for infectious cases, her remuneration will be augmented. Thi is the crux of the question, but it is a serious state of things that hundreds of nurses who have ministered to the sick and suffering should be so badly paid that they are unable to make any provision for the time when they are unable to work. If it is impossible to increase the salaries of the nurses, cannot the hospital authorities at least help to secure them the benefits to be derived from belonging to the Royal National Pension Fund?

### THE NURSES' CLUB IN DUBLIN.

In consequence of the rooms selected for the Nurses' Club in Dublin being adjacent to a smoking-room, and, in fact, on the same premises, two or three principal matrons and their nurses have withdrawn their support. This is an unpromising start, and the Committee should have taken care to choose rooms in a position to which no objection could be urged. But perhaps the selection made is not irrevocable.

### THE DEMAND FOR JUBILEE NURSES.

In the interesting annual report of the Portmadoc District Nursing Association, the question of holidays is touched upon. "There is no doubt," says the honorary secretary, "that the ideal arrangement for the nurse's holidays would be that a Jubilee nurse should be free in the summer months to undertake holiday duty. Such a nurse could be employed during the winter in towns and districts where the work is found too heavy for the permanent staff from the middle of September to the middle of April." Unfortunately, she goes on to say, this plan is not likely to be realised, "because the demand for Jubilee nurses at present far exceeds the supply." This is a statement to be noted by nurses who are on the look-out for a suitable sphere of work. The conditions on which admission can be obtained to the ranks of Queen's nurses have been lately set forth in the Nursing Mirror, and the high appreciation of the manner in which those already belonging to them discharge their duties, as shown in the report of the Portmadoc and other associations, should tend to encourage others to avail themselves of an admitted opening for ability and devotion.

### AN ANSWER TO NURSE DOWLING'S CHARGES.

The reverse sustained by Miss Dowling has made her bitter. We admit that she has a right to be heard, but the letter from her which, at her request, is published in another column, will not, we fear, do her any service. Whatever may have been her own experience, her general assertions are too obviously unjust and absurd to need refutation. "Hospital life to the poor probationer," she says, "is a veritable treadmill, and the life in private nursing homes may be compared in many respects to life in a prison. Matrons sit in easy chairs and read novels, and every nurse on the staff they employ is made to do the work of three nurses. There is no pity for a nurse when she is ill, no welcome for her when she returns sad and weary from deathbed scenes, no mercy for her if she protests, as I did, against overwork and harsh and unjust treatment." A striking practical commentary on statements of this kind is supplied by the matron of St. Mary's Hospital. Miss Medill tells our representative that the nurses who have been trained at St. Mary's and are now engaged in private work often ask to be allowed to return to the hospital to spend two or three months in one of the wards, and regard permission as a privilege. The matron says that she is always glad to have them back again providing they are well and strong, and that in the summer holidays the arrangement is particularly useful. But if they had been badly treated during the period of their training, they would obviously take very good care, when once they were quit of the hospital, not to enter its doors again.

### THE OLDHAM GUARDIANS AND THEIR PROBATIONERS.

It will not be surprising if under existing conditions the Oldham Board of Guardians experience considerable difficulty in obtaining candidates for training at the workhouse infirmary. Reports of meetings and letters in the local papers show that the position of affairs is very unsatisfactory. The guardians are extremely irate with Dr. Young, the non-resident medical officer, because he refuses to sign the certificates of four probationers who were admitted without his knowledge, previous candidates having always appeared before the superintendent nurse and himself that they might be jointly assured of their eligibility. But he advises that the custom of signing certificates should, in future, be discontinued, mainly because there is "no house surgeon, no regular instruction or examination held, such as is now generally recognised to be essential in the case of certificated nurses." This advice seems most unpalatable to the guardians, who are, of course, free to reject it, but are not free to shower vulgar abuse upon the adviser and those who side with him. We are exceedingly sorry for the four probationers who will apparently fail to get their certificates signed by Dr. Young, more especially as several nurses who were trained at the Oldham Infirmary are now in possession of good posts.

### THE CUMBERLAND NURSING ASSOCIATION.

Referring to a note in our issue of November 3 on the annual report of the Cumberland Nursing Association, according to which it seemed that of the 25 nurses working in connection with it, ony six were fully-trained, the Superintendent-Secretary wishes it to be known that the rule of the Association has been "that no village nurse shall have had less than six months' training, not that she shall have had no more than six;" and she adds that "two of the village nurses hold certificates for three years' general training." The Superintendent-Secretary says that "to let it be supposed that the title 'village nurse' is equivalent to that of 'uncertificated nurse' would be injurious to the cause of the association," and she states that "there are still many fully-trained nurses who will work in scattered villages accepting a salary of £70 with uniform and bicycle provided." Undoubtedly; and we agree with our correspondent that the title of a village nurse does not necessarily denote lack of training. But the President's demand for "good strong girls" as nurses certainly did not convey the impression that three years' training is considered of the greatest importance by the Cumberland Nursing Association. However, we are glad to learn that the desire of the Association is to have as many fully-trained nurses who have also had six months' special district nursing as funds will permit; and to receive the assurance of the Superintendent-Secretary "that suitable women with even as little as six months' training can do very much towards supplying the nursing needs of these scattered rural parishes."

### KENDAL HOUSE NURSING ASSOCIATION.

The annual report of the Kendal Nursing Association has been forwarded to us. We regret to learn therefrom that the committee can no longer afford to pay an assistant nurse for the winter months. The cost, about £40, was formerly defrayed by special efforts, such as a garden sale, a bicycle parade, or some form of entertainment; but as the Association has failed this year to receive such help they have to do the best they can with one nurse. Such an evident need ought to be met by increase of regular subscriptions.

### ACROSS THE SEAS.

From Australia comes a letter expressing the gratification experienced by the lady superintendent of the Melbourne Hospital on reading in our columns Miss Gardner's "In Memoriam" of Miss Hanan, the late matron of Crumpsall. Miss Farquharson writes:—"I am proud to have been her pupil, at Crumpsall Infirmary, and thankful also for the great help she has been to me by example in my superintendentship. . . . The nursing world has indeed experienced a loss by her death."

### A NEW DEPARTURE BY THE LOCAL GOVERNMENT BOARD.

The Clerk to the Farnham Board of Guardians has received from the Local Government Board an order transferring certain duties of the Master and Matron to the Superintendent Nurse. Article 1 of the order runs thus:—"It shall be the duty of the Superintendent Nurse to visit each of the sick and lying-in wards of the workhouse daily and to see that such wards have been duly cleansed and are properly warmed and ventilated, and also that all such arrangements are made as may be necessary for the proper care of and attendance upon the inmates both by day and during the night." Article 2 is as follows:—"The duty of making morning and nightly visits to the sick and lying-in wards of the workhouse shall cease to be part of the duties of the Master or Matron of the workhouse, as the case may be; but, except to this extent, nothing in this order shall affect the duties of the Master or Matron so far as such duties relate to the general control of the workhouse." The orders, we understand, only refer so far to Farnham and Basingstoke infirmaries, and are experimental.

### EPSOM UNION INFIRMARY.

The schemes for training probationers at the Epsom Workhouse Infirmary devised some three years since by the medical officer and matron has worked well, and last week certificates were granted, on the report of an independent examiner—a member of St. Thomas's staff—to those probationers whose term of service, conduct, and attainment were all satisfactory. The method of training was described by the examiner as being sound and practical. One probationer has obtained her L.O.S. certificate while at Epsom. It may be added that for four vacancies for probationers there were sixty applications, and that the "home life" of the probationers has been as carefully studied as their professional training.

### SHORT ITEMS.

The additional building of the Metropolitan Nursing Association for training and providing nurses for the sick poor, in Bloomsbury Square, will be opened on Tuesday afternoon.—The annual ball in connection with the Blackburn District Nursing Association will take place on Friday, January 11th, 1901.—A very successful concert was held in "David Wise Ward" of the National Hospital for the Paralysed and Epileptic, Queen's Square, on Saturday November 3rd. A capital programme was artistically carried out by well-known artists, who gave their services voluntarily, much to the delight of the patients and staff who were able to be present.—The Mothers' Seaside Convalescent Home, Herne Bay, has, we regret to learn, been permanently closed for the want of funds to carry it on. It was established in 1890 by the late Sir Rawson Rawson in memory of his daughter, Miss Frances Rawson, for the reception of mothers with or without infants and young children, and women and girls from all parts of the kingdom. Many letters of gratitude have from time to time been received by the lady superintendent, testifying to the appreciation of the advantages afforded to the inmates.—The funeral of Miss Ethel Fisher, the founder of the Taunton District Nursing Association, and for many years the devoted lady superintendent, took place last week. Miss Fisher acted in her youth as honorary nurse at the Taunton and Somerset Hospital, and was greatly esteemed by all classes.

### Lectures on Medicine to Nurses.

By H. A. LATIMER, M.D. (Dunelm), M.R.C.S. Eng., L.S.A.Lond.; Consulting Surgeon, Swansea Hospital; Past President of the South Wales and Monmouthshire Branch of Brit. Med. Assoc.; Past President of the Swansea Medical Society; Hon. Life Member of the St. John Ambulance Association, &c.

### FEBRICULA: SOURCES OF SIMPLE FEVER. REFLEX ACTION ILLUSTRATED.

AFTER a reference to one more characteristic change in the face, caused by disease, I will pass from this subject. I allude to hectic fever—a complaint accompanying suppurative diseases, and one which is a frequent accompaniment of phthisis of the lungs. Here the sufferer presents a characteristic appearance, the eyes being bright and glistening, and the skin covering the cheek-bones assuming a vivid pink flush. Such a face is an attractive one to the superficial observer, but to the trained and skilled onlooker it is only too evident that it is the outward and visible sign of a grave disease which is wearing out the sufferer's health.

I have told you in a previous lecture that fevers are in the main divided into two great classes according to their causes, being, in the one case, the result of infections by specific organisms, and, in the other, being secondary to inflammations of organs within the body. The simplest form of them that we meet with is called febricula, or simple continued fever. I note that in modern text-books of medicine this name has dropped out of the classification of febrile disease, and I cannot but agree with the wisdom of the writers who have discarded it from their list; for, as a pathological entity febricula, or lesser fever (as is the meaning of the term), has no existence. But the name is a useful one, none the less, and if we retain it with a clear and definite notion in our minds of its true meaning, and of its limitations as marking a feverish condition due to the irritation of the system by various temporary disturbances, can do no harm to the cause of scientific medicine. Such a fever is caused by a variety of disturbances to health, and may be the accompaniment of systemic worry set up by disorders of digestion, by the irritation induced by worms, by over-exposure to the sun's heat, by teething, or by mere nervous excitements. As all such disturbances are peculiarly prone to occur during the period of childhood, this feverish state is especially one of that time of life, I would not have it thought that it is only then that we meet with it, but I shall certainly be right in saying that for one case occurring in adult life we shall meet with three or four dozen cases in young childhood. There is a reason for this as in all other things. The nervous system of children is far more delicate and sensitive than is the nervous system of adults. Then growth has been completed, and maintenance of the body, at its full development, is all that has to be done; but in the earlier years of life the child has to grow in stature, and development of organs has to take place, hand in hand with the full exercise of their functions. I find it to be most difficult to get people to understand the use of the nervous system, and oftentimes wish that they knew something about the elementary truths of physiology—that science which regards the general laws of life. They fail to see why a disturbance of the nervous system should produce disease, or why an impairment of the working of an organ in the body should result in a disturbance of the nervous system. If they would grasp the fact that this brain and spinal cord, with the nerves which flow from them, and the sympathetic system of nerves, which are all interdependent, are the carriers of that vital force which we call nerve force, and which is the mainspring of life, they would see how health can only be maintained by their perfect integrity. If they could only realise that the body is built up by the influence of nerve force carried by delicate nerve-threads to every minute cell of it, and that by means of this system each cell is in communication with a receiving nerve centre which telegraphs back messages in response to irritations which have reached it, when that cell has been disturbed in any way, they would have made a great step in knowledge of health and of disease. Let it be understood, then, that no movement of any part in the body, and no performance of any functional act by any organ within it, can take place without a message being sent from nerve-centres to the appropriate part concerned in the operation: and let it be further understood that the greater number of these acts are in response to stimulations sent by some routes to the centres, and with orders telegraphed back through other routes to the parts which are concerned. This is the great law of "reflex action" which was formulated by that distinguished physiologist, Dr. Marshall Hall. This law of action in response to stimulation obtains everywhere in the organism, and I propose to cite a few common-place instances of it to bring home to you a realisation of the fact. You know how quickly an eye becomes "blood-shot" when some foreign body has alighted upon its surface. A piece of grit, say from an engine, strikes it and remains upon it, and immediately there is pain in the eye, there is profuse watering of its surface, and a number of minute blood-vessels, of whose mere existence you knew nothing beforehand, fill with blood, and so set up a local active congestion at the irritated part. What has happened has been this: the sensitive nerve-ends of the eye, irritated by the foreign body, have telegraphed back to their centre a message to that effect; immediately messages have been telegraphed back to the lachrymal gland, which manufactures tears, to send a flow of these over the eye to soothe it and to wash away the offender; and the vessel dilating nerves have also been ordered to work so that the capillary arteries become distended with an unusual amount of blood. This example of reflex action, one culled from many others that I might cite, is a pathological one—that is, one connected with disease. Now let me quote a physiological example of an every-day occurrence. The acts of digestion of foods are performed by the bringing into play of the same operations. For, food having been introduced into the digestive track, all along its route reflex nerve action comes into play, and the various glands which pour out digestive juices proceed to do so on stimulation to that effect. One more example and I will pass on with my subject. This time I will cite a mental operation: I will take the familiar instance of the "mouth watering" when sight or smell have been excited at the thought of a savoury meal being forthcoming. Here is quite a complex operation, and the stimulation of the salivary glands has been truly reflex in response to stimulations conveyed to brain centres which are connected by communicating nerve channels—with the optic and olfactory nerves on the one hand and the salivary glands on the other. The great facts which you are called upon to grasp in connection with the rôle of the nervous system are that, by a multitude of connecting links, divers organs and nerve centres are brought into communication with each other, and that the irritation of organs results in their doing their appointed work faultily, with the result that the body generally suffers from deranged health. Looked at from this point of view you will easily understand how irritations of the digestive organs, or irritations of the nerves from the pain of teething in childhood, will set up a disturbance in the central system, and so cause fever. For many of these irritations are ones which affect mucous membranes—that is, inner skins—by causing active congestions of them to occur (just as the eye becomes congested on its surface when irritated by a foreign body), and whenever you have an abiding congestion of a mucous membrane you have the process set up known as catarrh. Thus many cases of simple continued fever are merely ones of reactionary functional disturbance due to passing irritants, which, when removed, allow of a return of the disturbed part to its normal state of health, with a concurrent decline of the fever which has been the accompaniment of the whole process. These points I must amplify in my next lecture.

*(To be continued.)*

### The Nurses of St. Mary's Hospital.

BY OUR COMMISSIONER.

### A CHAT WITH THE MATRON.

It is well known in the nursing world that no radical alteration has been made in the regulations for the training of nurses at St. Mary's Hospital except binding for four years, and when I called to see the matron the other day the first question I asked was "Why did you extend the term of training to four years?"

"We do not call it an extension of the term of training," replied Miss Medill, "but according to the regulations which came into operation in April the probationer who has finished her training in three years and earned her certificate is required before she receives it to serve the hospital for another year as staff nurse."

### THE ADVANTAGES OF THE FOUR-YEARS SYSTEM.

"This change was made in the interests of the hospital, and because we lost so many of our nurses at the end of the three years. The outlets for them are now so numerous and so many go in for private nursing. The Army Reserve has also depleted the ranks of the nurses. I do not at all blame them for doing the best they can for themselves. People look on nursing sentimentally, but, after all, it is a profession, and it is necessary for the hospital authorities to hold out every possible inducement to their nurses to remain."

"Then you consider that the extra year is beneficial to the nurses?"

"Certainly, The fourth year is, I think, quite invaluable to them. After the wear and tear of theoretical instruction is over, with increased knowledge, they can go so much better with the whole subject. In addition to the certificate for three years' training, a further certificate is given at the end of the fourth year."

### THE RESULT.

"Has the alteration had any effect upon the number of certificates for admission?"

"None whatever. I confess that this has surprised me a little. When the matter was under consideration the committee asked me if I thought that it would, and I told them candidly I did not know. But, as a matter of fact, no one has offered any objection to the four years. One reason for this doubtless is because it is becoming general, as candidates who apply to other hospitals soon find out. I make a practice of encouraging candidates for admission at St. Mary's to try all round. The result is that if they come here they settle down all the better."

"Of course, applications are first made to you?"

"Yes, I take up their credentials as to age, which must be between 23 and 35, character and education, and see if I think they are personally suitable. The medical superintendent next examines them as to health and physical fitness. This has been done for a number of years, and is all the more necessary now that the period of service is four years. If this examination proves satisfactory, the names are submitted to the committee."

"Then the matron does actually make the appointments?"

"No; I select, but the committee approve, and no candidate can be admitted without their sanction."

### THREE MONTHS' TRIAL.

"Another change we made in April," continued the matron, "was to alter the trial period of service in the wards from one to three months. I had for a long time felt the disadvantage of the one-month plan. A month is really too short for a fair trial of capacity and health. Thus a candidate may do very well for the first two or three weeks, and not so well the next two or three. Practically, the matter is settled one way or the other before the end of three months. This change has not diminished the number of candidates."

"Do you have the same class of applicants as you had before April?"

"Yes. I do not see any difference. Most of the applicants belong to the better class. It is, of course, requisite that a girl should be strong enough physically to take her part in the manual labour, but girls, where education has been liberal, with a larger outlook, are more likely to succeed as nurses."

### THE COURSE OF INSTRUCTION.

"I should like to know what you do in respect to instruction?"

"There is at present no entrance examination, but when a candidate is admitted we expect her to have an elementary knowledge of anatomy and physiology. At the end of the first year's training probationers are submitted to an examination in subjects taught them during that period. If they fail to attain a reasonable standard they leave. But a girl of average intelligence and of industry should pass. The difficulty is that they have to pursue simultaneously the practical as well as the theoretical work. After passing the first examination probationers receive regular courses of instruction from members of the hospital staff in medical, surgical, and obstetric nursing Their knowledge is again tested at the conclusion of these courses, and they do not obtain certificates until they have passed this examination also."

"How about operations?"

"There is a sister in charge of the operation theatre, with a staff nurse and a probationer during the day, and a nurse on duty at night. In the small obstetric theatre the ward sister and her staff attend all the operations."

### OFF-DUTY AND HOLIDAYS.

"What is the extent of the nursing staff, and what are their hours off duty?"

"There are fourteen sisters including the night superintendent, and seventy-five nurses. The probationers have two hours off duty daily: one Sunday about four hours in the morning, and the other from 3 to 10. The staff nurses get two evenings in the week free, one from 5 to 10, and the other from 7 to 10, with the same off duty as the probationers on Sunday. The evening leave of the sisters is extended to 10.30, and they also get two hours off every Saturday. As sisters' evening falls on Saturday every two weeks, this gives them practically a half-day once a fortnight. They have a whole day every month as well. As to holidays, the sisters have a calendar month, and the staff nurses and probationers have three weeks."

### THE ACCOMMODATION FOR NURSES.

"How are you off as to accommodation?"

"In our new scheme, designed some years ago, provision is made for a very nice nursing home in the hospital. It is proposed to put the nurses at the top of the building. Unfortunately, all that has been done so far in respect to carrying out the scheme is that we have roofed in the out-patients' department. We are collecting money for the new building, which is badly wanted, and some day, I dare say, we shall start all in a hurry. Meanwhile, we do the best we can, and are pretty comfortable."

"You have a house outside?"

"We have two now in Cambridge Place. The second was taken lately, and we are able to provide for some of the day nurses. The first house is occupied exclusively by the night staff. There is a good sitting-room, and the bedrooms are of fair size. It is rather an advantage to the night nurses to sleep out."

"I suppose you anticipate it will be an advantage to the hospital when you get a frontage in Praed Street?"

THE RECREATION QUESTION.

"Unquestionably. Speaking of advantages, we find that it is one to be so near Hyde Park. Many candidates have told me that they like to come to St. Mary's because of its proximity to the Park. In the matter of recreation, late leave is given once a month, and if any one sends the nurses tickets for the theatre, or for a concert, I make special arrangements to enable them to go. We frequently have tickets sent."

"And you have occasional entertainments in the hospital?"

"Two entertainments are given at Christmas. The nurses are provided with a piano and with books."

PRIVATE NURSING.

"Should you like to have a private nursing staff?"

"I should, for I consider it a very valuable adjunct, though in existing circumstances, it is impossible. But I am sure it is a good thing for hospitals to send out nurses. On the one hand that they are attached to the hospital is a guarantee of their efficiency, and on the other nurses have facilities for rubbing up their knowledge. We often have nurses who were trained here, and are now in private work, return to the hospital for holiday duty. They consider that it is a privilege."

"Are you glad to have them back?"

"Always, so long as they are well and strong. They usually stay for two or three months, and are employed in medical or surgical work, according to preference. They receive staff nurse's salary, and we find that in the summer holidays the arrangement is useful to us. I get letters about March asking if they can come; and as a rule the nurse who wants to return is just the kind of nurse we like to have."

THE PAYING PROBATIONERS.

"How many paying probationers do you receive?"

"Ten. They have a year's instruction in nursing, and many of them have had previous training in a children's hospital. Their duties are similar to those of the ordinary probationers in their first year."

"Do they ever stay on?"

"Often. In such cases they proceed on the same terms as the others. I have frequently had paying probationers who turned out most valuable nurses. They get no certificate for the year, but merely a voucher for work done. If they decide to remain, they have to pass the committee just as if they had come in fresh altogether."

"One more question. How many of St. Mary's nurses are in South Africa?"

"Four sisters went out—one has since returned to England invalided after enteric fever. We are keeping their places open for them here. They were precisely the right sort of women for the work. I do not care to discuss the question of the reorganisation of the Army nursing service, but I am convinced of the vital importance of every care being taken to secure a full staff of suitable nurses in the event of another war."

### The Scottish Red Cross Hospital.

A RETROSPECT BY A SISTER.

THE Scottish National Red Cross Hospital was stationed at Kroonstad. The nursing staff consisted of 34 sisters, under the superintendence of Miss Shannon, night superintendent of the Glasgow Western Infirmary, who had been granted leave of absence for six months in order to fill that post.

The hospital contained 520 beds. The work of nursing was lightened owing to the fact that we had first-class orderlies (medical students), who assisted the sisters. The patients were housed in tortoise tents and in döcker huts. The tents held 10 beds each and were all floored. The döcker huts contained 16 beds. These huts were made of papier-mâché, were very light of transit, and easily put up. They stood a few feet off the ground; they had six windows in either side, a door at either end and one at either side of the hut, besides no end of ventilators. Therefore they could always be well aired. In rain or sand storm they were splendid; in the severe weather they were much warmer than the tents.

THE PROVISION FOR THE PATIENTS.

The patients had iron bedsteads with spring mattresses, splendid bedding—sheets, blankets, &c., in plenty. The appliances for the use of the patients were also plentiful. One thing the soldiers did enjoy, and that was a bath. There was one for each tent or hut. Accordingly therefore every man was bathed on admission if at all fit. Some excellent work was done; surgical cases, many of them most interesting, and a full share of enteric, dysentery, rheumatism, &c., &c.

THE HOURS OF DUTY.

The sisters went on duty in the morning at 8.30 A.M., lunched at 1 P.M., went back to wards, then were off duty until 5 P.M., dinner 6.30 P.M., returned to wards for doctor's second visit, finally off duty 9 P.M.

There was an orderly sister on duty each day. She was responsible for the cases in the afternoon when the other sisters were off duty. When there was a really bad case a special night and day nurse were put on, so the patient was never left a minute. The sisters took night duty in rotation; their number varied according to the cases. They went on duty at 9.30 P.M.—the orderly sister for the day stayed on until they came—and were off the following morning at 8.30. Their time on night duty was a fortnight. In June and July the nights were very cold; the sisters had a stove (coal) in their mess tent which materially added to their comfort.

THE SLEEPING ACCOMMODATION AND MEALS.

An iron hut containing bedrooms and special rooms for the superintendent was sent out—it could not be sent up country at first, so it was never occupied. We slept in tents, three single and all the others in double tents—square, and bell-shaped. The interior of these tents was very comfortable. We had nice beds and bed-linen. Then there was a chest of drawers for each sister with a most ingenious arrangement for basin and jug which was quite an ornament and could play the part of a desk. There was a floor to each tent and each nurse had a gay-coloured mat by the side of her bed. Each tent was also supplied with a small bath, large hot-water can, &c. A Kaffir girl looked after the emptying of the slops, &c. Orderlies washed the floors and carried the water, emptied the baths, &c. Our personal washing was done by Kaffir women. The washing for the hospital was done by men.

The nurses had a döcker hut as a mess tent; it was most admirably arranged. Our table appointments were excellent, and when I add that only one of the sisters was ill—she had a very slight attack of enteric—I am sure it was due in a great measure to our food being well looked after and well cooked.

THE PASSAGE HOME.

Six sisters and myself nursed in the several hospitals, Bloemfontein, until our own hospital was erected. Then seven sisters came back with me on the ss. *Trojan*. We had thirty-seven soldiers, most of them convalescent, though there were four spinal cases, one or two phthisical cases (pretty far gone), also some cases of dysentery. Still, everyone improved on the passage home. We had twelve convalescent officers. Fortunately, we had a very good passage, and we all thoroughly enjoyed the novelty of working on board a hospital ship.

The Scottish National Hospital has been handed over to Government. I believe it retains its name. Eight sisters are staying in South Africa, and I understand that the others are returning very shortly.

### The Local Government Board AND The Crisis at Croydon Infirmary.

As already reported in the NURSING MIRROR, difficulties and disagreements have arisen between the Croydon Guardians and the Matron of the infirmary. The facts briefly are these:—The Matron refused to sign the certificates of three probationers at the close of their three years' service, on the ground that they were incompetent. The Medical Officer held a different view of their proficiency. The Board of Guardians as an outcome of the trouble took the charge and instruction of the probationers from the Matron, so confining her to the more domestic duties of her office. Miss Julian thereupon appealed to the Local Government Board. She said she could not believe that the Croydon Guardians had the right to depose her from her recognised position by the Local Government Board as superintendent of the nurses, or to determine that she should sign no more certificates of training. She further urged that the action of the Croydon Guardians rendered the Croydon Infirmary no longer a training school for nurses, and that this was a monstrous injustice to the present staff of nurses. No reason, she stated, had been assigned by the Guardians for this drastic treatment. Presumably it was because she had refused to sign the certificates of three women who were in her opinion unworthy of the name of nurses, and whose general conduct was eminently unsatisfactory. The next step in the controversy was that the Croydon Guardians were asked by the Local Government Board for their statement of the facts. The Clerk's letter from the Croydon Board set forth that the Croydon Guardians had had serious cause to be dissatisfied with the manner in which Miss Julian had exercised control over the nurses, and, as her general want of discretion and tact in dealing with the officials had led to more than one inquiry by the Infirmary Visiting Committee, the Guardians had been obliged to disconnect her entirely from the nursing staff, and the Guardians submitted that they were perfectly justified in adopting the course they had done. In addition to these communications, the Local Government Board were put in possession of a voluminous correspondence between the Matron and the Clerk to the Board, the contents of which were not disclosed in public.

At a meeting of the Croydon Guardians held on Tuesday the matter was again before the Croydon Board, owing to the receipt of a letter from the Local Government Board which seemed to favour the Matron's view. It was dated November 1st, and stated that the Board are not in possession of sufficient information to enable them to express any opinion as to the course adopted by the Guardians, but in the interests of the administration of the Infirmary they cannot but regret the state of things indicated by the correspondence which has taken place on the subject. The arrangements for the supervision of the nursing staff and nursery duties, as well as for the training of probationers, are matters of great importance, and in this connection the Board would specially direct the Guardians' attention to the petition signed by twenty-two nurses of the Infirmary, a copy of which was enclosed in the Matron's communication to the Board dated September 25th last. The Board desired to be informed of the result of the further consideration of the whole subject by the Guardians. This letter having been read at the meeting, Mr. Shirley, Chairman of the Infirmary Committee, moved that it be referred to that committee. He asked whether the Guardians had been favoured with a copy of the nurses' petition furnished to the Local Government Board? The Chairman said the Guardians had not been furnished with a copy. Mr. Morland said the Infirmary Committee ought to have a copy of the petition before them when considering the matter. Mr. Shirley observed that this "round robin" by the nurses was not presented to the Infirmary Committee. The motion referring the whole question to the Infirmary Committee for reconsideration was agreed to unanimously. The petition of the twenty-two nurses sets forth a resolution passed by them to the effect that a certificate without the matron's signature is of no value whatever in the nursing profession, and that they hoped that the Guardians, who could never have realised how much the new regulations meant to the nursing staff (both past and present), would again give the matter their serious and careful consideration.

### Appointments.

HULL HOSPITAL FOR WOMEN AND ORTHOPÆDIC.—Miss A. E. Simpson has been appointed matron. She was trained at the Royal Infirmary, Hull, where she has since been nurse. She has also been assistant matron at the Convalescent Home, Withernsea.

BUTE AND ARGYLL ASYLUM, LOCHGILPHEAD, N.B.—Miss Mary Murray has been appointed matron. She was trained at the Royal Infirmary, Glasgow, afterwards holding the post of staff nurse in the same institution for three years. For the last five years she has worked as Queen's Nurse in Musselburgh, N.B.

COTTAGE HOSPITAL, OBAN.—Miss Alice Tweedale Brierley has been appointed nurse matron. She was trained at Guy's Hospital, and has since been nurse at the Hertford Hospital, Paris, and the Greek Hospital, Alexandria, and matron for two and a-half years at Tetbury Cottage Hospital. Miss Brierley holds the L.O.S. certificate.

LADY HOZIER CONVALESCENT HOME, LANARK.—Miss E. More has been appointed matron. She was trained at the Western Infirmary, Glasgow, where she afterwards filled the position of dispensary sister for four years, and assistant matron for two and a-half. A short interval during the early part of her hospital career was spent in private nursing in the South of France.

ABINGDON ISOLATION HOSPITAL.—Miss Ellen Glover has been appointed matron. She was trained at the Queen's Hospital, Birmingham, and has since been staff nurse at Brompton Consumption Hospital; sister at the General Hospital, Birmingham; and charge nurse at the North-Western Hospital of the Metropolitan Asylums Board. She has also been engaged in district nursing at Edinburgh and in Kent.

### Minor Appointments.

PLYMOUTH WORKHOUSE.—Miss Annie Fisher has been appointed assistant nurse. She is a member of the Meath Workhouse Nursing Association, and was trained at St. Peter's, Maybury Hill, Woking.

BUCKLOW UNION, KNUTSFORD.—Miss Annie Thomas has been appointed assistant nurse. She was trained by the Meath Workhouse Nursing Association at St. Peter's, Kilburn.

ROYAL BERKSHIRE HOSPITAL, READING.—Miss C. Spackman has been appointed sister. She was trained at the Brompton Hospital, and has since been staff nurse at University College Hospital.

ELTHAM COTTAGE HOSPITAL.—Miss Hanson has been appointed staff nurse. She was trained at Bristol Royal Infirmary for three years, has since been charge nurse in a surgical home, and has also done district and private work.

ST. SAVIOUR'S INFIRMARY, EAST DULWICH GREEN.—Miss Millicent Violet Ryott has been appointed sister. She was trained at the Royal Infirmary, Bradford, and has since been sister at the Royal Infirmary, Ryde.

### To Nurses.

WE invite contributions from any of our readers, and shall be glad to pay for "Notes on News from the Nursing World," or for articles describing nursing experiences, or dealing with any nursing question from an original point of view. The minimum payment for contributions is 5s., but we welcome interesting contributions of a column, or a page, in length. It may be added that notices of entertainments, presentations, and deaths are not paid for, but, of course, we are always glad to receive them. All rejected manuscripts are returned in due course, and all payments for manuscripts used are made as early as possible at the beginning of each quarter.

### Nursing in Workhouse Infirmaries.

### THE NURSING ACTS OF 1897—HOUSEMAIDS EMPLOYED AS NURSES.

THE question of nursing is dealt with by most of the inspectors of the Local Government Board in the annual report for 1899-1900.

### THE HOME COUNTIES.

Mr. J. W. Preston, the inspector of the district comprising the Union counties of Bedford, Hertford, Huntingdon, Middlesex, Northampton, and Cambridge—Wisbech excepted —report that "the care of the sick and aged is receiving more attention by Guardians generally. Nurses of training and experience are gradually taking the places formerly occupied by inmates. The adequate nursing of the sick and aged in small rural workhouses is full of difficulty."

### EAST ANGLIA.

Mr. Philip Bagenal, inspector for the district comprising the Union counties of Norfolk and Suffolk and parts of Essex and Cambridge, says:—

"The difficulty in obtaining and retaining the services of nurses for workhouses continues to increase. Notwithstanding, the results of the Nursing Order of 1897 has effected a great improvement in the nursing arrangements of this district. From information supplied to me by the various unions I am able to give an approximate idea of the number of patients per nurse. On December 31st, 1899, Wisbech had one nurse, one assistant nurse; 96 patients; average, 48 patients per nurse. Essex had 7 superintendent nurses, 23 nurses, 11 assistant nurses, 6 probationers; total, 47; patients, 1,030; average, 22 patients per nurse. Norfolk had 5 superintendent nurses, 26 nurses, 25 assistant nurses, 11 probationers; total, 67; 1,136 patients; average, 17 patients per nurse. Suffolk had two superintendent nurses, 21 nurses, 6 assistant nurses, 2 probationers; total, 31; 731 patients; average, 24 patients per nurse. The average number of patients per nurse for the whole district works out, roughly, at 20. It must be remembered, however, that the term 'patients' includes all names upon the medical relief book, and a large number of these are not bedridden, and often not even in the sick-wards. It has been laid down by a competent authority that, speaking generally, one nurse, under favourable conditions, may look after 30 patients; but she can only do so by day or by night, not both by day and by night. Therefore the 30 patients require two nurses, one by day and one by night. In other words, as a general rule, not less than one nurse to 15 patients is required for the class of cases usually found in workhouse sick-wards. Even this proportion is not sufficient when the sick-wards are scattered about, and heavy cases unduly preponderate. Guardians are apt to overlook the necessity of night nursing. It is just as much a necessity as efficient day nursing."

### THE SOUTH OF ENGLAND.

Mr. Baldwyn Fleming, inspector for the district comprising the Union counties of Dorset and Southampton and parts of Wilts and Surrey, reports:—

"The nursing arrangements follow the character of the Boards of Guardians. Gradual improvement may be noticed, and in some unions a great deal has been done to secure proper attendance upon the sick. The matter depends upon the disposition of the Guardians. If they will pay and treat nurses well, good nurses will go to them. Good nurses will not go where the remuneration is insufficient, and where the necessary arrangements for the care of their patients and for their own comfort are absent. It is satisfactory to state that due provision for the nurses has been undertaken at Hartley Wintney, Christchurch, Petersfield, and Alverstoke, in addition to places previously mentioned. Nursing appointments have proceeded in fair proportion, but as yet it cannot be said, with any approach to truth, that the use of paupers in nursing duties has been wholly discontinued."

### THE MIDLANDS.

Mr. E. B. Wetherhed, inspector for the district comprising the Union county of Gloucester and parts of Hereford, Somerset, Stafford, Wilts, and Worcester, says:—

"I regret to have to report friction between masters and matrons on the one part, and nurses of the infirmaries on the other. This must have a bad effect on the general administration. I do not propose to discuss on which side the fault lies, but I do look forward to the day when, in workhouses of any size, the infirmaries will be detached, and greater responsibilities imposed on the medical officer and superintendent nurse. Even under the present circumstances I think that this friction could be to some extent avoided if, where there is a superintendent nurse, the responsibilities of the master and matron could be defined. I am not unfrequently asked what these duties are. Respective masters and matrons take different views, and the same remark applies to superintendent nurses. Arising out of this uncertainty some masters and matrons interfere more than others, and not always with tact. On the other hand, nurses sometimes appear not to realise that the master and matron have responsibilities with regard to the infirmary, and that they naturally desire to discharge them. Indeed, failure to do so might mean serious consequences to themselves. The employment of probationary nurses is on the increase, the object generally being to save expense in the way of salaries. Where there is a resident medical officer there can be no objection to the employment of probationers within reasonable numbers. A capable class of young women apply who look forward to rising in their profession. But where there is not a resident medical officer probationers cannot rise above the position of a charge nurse. They cannot qualify for the position of a superintendent nurse, and consequently it cannot be expected that the most capable young women, who desire to be trained as nurses, will apply under these, what I may call, restrictive possibilities for the future. The question therefore arises, whether it is desirable that probationers should be allowed unless under conditions which permit of their ultimately qualifying as superintendent nurses. If, however, the present system is to be continued, then I suggest that the number of probationers to be allowed, under the varying circumstances, should be defined. The practice, too, of putting young probationers on night duty by themselves is one which should be avoided. The night duties are often very important. It is true that the probationer can call one of the trained nurses; but this is not desirable, except in the event of emergency. One object of a night nurse is to allow the others to rest. There are also other reasons which might be urged. Finally, I think it undesirable that probationers should take night duty, except with a qualified nurse, and in no case unless they have served at least one year of their term of probation and arrived at a suitable age."

### OTHER PARTS OF THE MIDLANDS.

Mr. R. J. Dansey, inspector for the distict comprising the Union counties of Salop and Chester and parts of Hereford, Stafford, Worcester, and Derby, reports:—

"The arrangements as regards nursing in workhouses are gradually improving, though the progress is slower than I could wish, for, taking the whole district, the number of beds per nurse is about 20, although in some unions it is still as high as 30. In several of the country unions, however, where the average number of beds per nurse appears to be high, there are no infirm wards in the main building of the workhouses, and consequently infirm inmates are placed in the hospitals, although requiring no regular nursing. The chief obstacles in the way of better nursing continue to be inadequate accommodation for nurses and the rather limited supply of experienced nurses; but Guardians generally are showing increased interest in the nursing question, and much of this may, I think, be fairly traced to the influence of those ladies who act as Guardians or as visitors to workhouses. It is satisfactory to note that Guardians are also turning their attention to the nursing of the out-door poor, and that, while in a few unions district nurses have been appointed, it has become more general for Guardians to subscribe to local societies that undertake to provide nurses for the out-door poor."

### LANCASHIRE AND WESTMORLAND.

Mr. H. Jenner-Fust, inspector for the district comprising the Union counties of Lancashire and Westmorland and parts of Cumberland and the West Riding of Yorkshire, reports:—

"Comparing January 1st, 1900, with January 1st, 1899, there has been an increase of 280 in the number of the sick, and of 68 in the number of nurses employed, including superintendent nurses, assistant superintendents, charge nurses, assistant nurses, and probationers, the numbers for January 1st, 1900, being 9,364 sick and 732 nurses. There is also a fractional decrease in the number of patients per nurse. If, however, we take the five years since the return was first issued, we find that while the sick have increased in numbers by 534, or 6·0 per cent., the nurses during the same period have increased by 227, or 44·9 per cent., and the number of patients per nurse has diminished from 17·48 to 12·79. Of course as the number of nurses in proportion to the number of patients approximates more closely to what is considered adequate, the rate of increase which has been so marked during the last few years cannot be expected to continue; but as no less than eight, or possibly nine, entirely new workhouse infirmaries are likely to be opened in the course of the next few years, many of them on a large scale, a considerable addition to the total number of nurses employed may be confidently expected, as well as a further decrease, though perhaps a slight one, in the number of patients per nurse."

DURHAM AND NORTHUMBERLAND.

Mr. C. A. DAWSON, inspector for the district comprising the Union counties of Durham, Northumberland, the North Riding of Yorkshire, and parts of Cumberland, reports:—

"The nursing staffs, at any rate in the larger workhouses, are being slowly strengthened; but the same difficulty still exists in getting nurses who have had a thorough training, and I do not think that in this part of England the blame can be wholly laid on the salaries offered or the quarters provided. In very small workhouses, of which there are several in the North Riding of Yorkshire, there are few if any actually sick; and in these, as I have already stated in a former report, it is useless to expect trained nurses to take situations. In these small houses the best plan seems to be to give the matron sufficient assistance to enable her to give her attention to the sick-wards, and for the medical officer to exercise freely his power of requring the master to engage temporary nurses when additional nursing is required."

THE EAST AND WEST RIDING OF YORKSHIRE.

Mr. H. G. Kennedy, inspector of the district comprising the union county of the East Riding of Yorkshire and parts of the West Riding of Yorkshire, reports:—

I have had the supervision of the workhouses—44 in number—in this district for rather more than thirteen years. At the time of my first inspection the sick wards contained collectively 4,141 patients in charge of 98 nurses, or on an average not quite one nurse for 42 patients; whereas during my inspections in the past year I found collectively 5,398 patients in charge of 346 nurses, or, on an average, rather more than one nurse for 16 patients. This is, indeed, a vast improvement on the state of things that existed so recently as 1886-7. In 1887 there was not one night nurse employed throughout the East Riding, a district which comprises such important workhouses as Hull, Sculcoates, and York. There were three night nurses at Leeds, and one at Sheffield, which last workhouse contained more than 600 patients. That was all! At the present time there are nurses on night duty at the nineteen largest workhouses. I am not able to give the exact total of night nurses, because the number varies from time to time, but I am in a position to say that in 1837 the figure was 4, and in 1899 it was at least 59. The qualification for duty possessed by nurses has, as is well known, vastly improved during recent years, and the strength of the staff at the larger workhouses is being steadily increased. It must not, however, be assumed that this picture is all light and no shade. Take, for example, the following four workhouses, where the sick are left to take care of themselves at night, or, what is perhaps rather worse for those unfortunate patients who may need occasional attention, in charge of pauper helps, viz.:—

| | |
|---|---|
| Holbeck, with about ... ... ... | 58 cases. |
| Goole " ... ... ... | 52 " |
| Knaresboro' " ... ... ... | 46 " |
| Selby " ... ... ... | 32 " |

It is in vain for me to urge the guardians to appoint a night nurse in such instances as these, because the workhouse medical officer, even though I may have spoken to him on the matter, in his next report, which he makes regularly every half-year to the guardians, in answer to the printed question, 'Is the nursing satisfactorily performed?' replies affirmatively. Thus my mouth is closed; the guardians point out that their medical officer is satisfied, and there is therefore nothing more to be done. In an order dated August 6th, 1897, the Local Government Board made some most important alterations in the rules as to sick nursing in workhouses. It is, among other things, thereby provided that nursing by pauper inmates shall cease. Yet, now that I have had upwards of two years' experience of the working of this most important general order, I am convinced that pauper inmates cannot be prevented from performing nursing duties—that is, that the provisions of the order cannot be complied with wherever, as in a large number of moderate-sized workhouses, there is a two-storied detached infirmary in charge of a single nurse, who has to be at work by day and get up also for emergency duties at night. Such a nurse, it must be borne in mind, has half-a-day's leave every week (which is little enough) and a holiday once a year for a fortnight or ten days, during which period a paid substitute is not in every instance provided. Suppose that the nurse is engaged in dressing an ulcerated leg on the female side of such an infirmary, and that a male epileptic on the men's side falls down in a fit, Of course, the pauper wardsman will assist the epileptic, while perhaps somebody else runs to inform the nurse, who cannot for the moment leave her dressings. But to loose a man's neckcloth and put a pillow under his head when he is rolling about in a fit is "nursing;" no amount of argument can make it out to be mere "attendance." Other similar illustrations might be given to support my contention, but I think the one which I have just quoted is sufficient to make it clear that the general order on nursing in sick wards cannot be duly carried out where only one nurse has charge of a two-storeyed infirmary. Before I leave the subject of sick nursing in workhouses, I may perhaps add that country guardians are unfortunately often very penurious about nurses' salaries. Nurses are but human beings. They are often comparatively young, and, like other young persons, they prefer what is lively to what is quiet and dull. Hence in remote outlying places, where the nearest town is miles away, to offer a salary of £20 a year, as is not infrequently done, seems to me ridiculous if the object is to secure a competent nurse. It also appears to me not altogether economical, for, if the salary is too low, changes are frequent, and money is expended in advertising which would be employed with more advantage in paying an adequate stipend. There is also a serious side to this picture. No qualified nurse has responded to an offer of £20 a year; but, after advertising again, somebody who has been a respectable housemaid, and who expresses herself most willing to undertake the duties, and assures the guardians that she *feels herself* quite competent, *does* turn up. She is appointed—but what happens when she has got to work? How much suffering may her willing ignorance cause to the patients? These questions cannot be exactly answered, but I can refer to an instance where, as the doctor declared, the 'Nurse' had never seen a confinement case before, and 'had no idea what to do;' and I can quote another where the 'Nurse,' being told to take a temperature in the course of the evening, put the clinical thermometer, which the doctor had left with her, into the chamber utensil; and a third case where, in endeavouring to pass a catheter, she caused the death of the patient. The moral is that untrained housemaids at £20 a year are not effective understudies of the nurse's *rôle*.

NORTH AND SOUTH WALES.

Mr. F. T. Bircham, inspector for the district comprising North and South Wales and Monmouthshire, reports:—

"It is difficult to obtain the services of trained nurses for the numerous small workhouses in the district—there is not sufficient work to attract them, and in very few instances have they been appointed. But in the larger establishments matters in this respect are gradually, if slowly, improving, though perhaps not to the extent to be found in most English districts."

### The Nurse in Hot Climates.

By EDWARD HENDERSON, M.D., F.R.C.S. Edin., late Surgeon, General Hospital, Shanghai.

(*Continued from page* 78.)

*Beef Juice and Beef Mince.*—The preparation of beef juice and beef mince is a matter which the nurse should thoroughly understand. To force the juice from meat in sufficient quantity a special press is needed, and this, along with a mincer for the preparation of the mince, should be part of the plant of all hospitals where cases of the kind under consideration are received. The juice of raw meat is occasionally prescribed, but is generally disliked by the patients; theoretically it may be more nutritious and possibly more easily digested, but in practice will be found little superior to the juice expressed from underdone steak. A pound of underdone steak freed from fat and skin, when cut up and put in the press, should yield a small coffee cupful of juice, and this is about the quantity needed at each meal—given about every two hours during the day. Along with beef juice a thin slice of well-toasted bread is usually allowed. The stools of patients undergoing treatment by beef juice are dark brown in colour, and of course scanty. The directions given for the preparation of beef mince in the so-called "Salisbury Cure" can scarcely be improved on, and should be carefully followed; in carrying these out the personal superintendence of the nurse will at first be required. The mincer recommended is the one known as the "Enterprise." The beef is either used raw, or browned quickly on both sides in a frying pan over a clear fire; meat intended to be cooked before it is minced should be cut in slices about an inch thick. The cooked or raw meat, freed from fat and skin, is cut in strips and passed through the mincer from three to five times. It is then beaten up with water in an enamelled saucepan with a wooden spoon until reduced to a smooth paste; water should be in the proportion of a teaspoonful to an ounce of meat; a little more warm water may be added if the mince in cooking becomes too dry. The cooking is done at a gentle heat on the side of the range, stirring all the time and raising the mince from the bottom of the pan, which should never be allowed to become so hot that it cannot be touched with the finger. If the mince smokes the pan must be at once raised. Raw meat takes about an hour to cook in this way, the meat from the frying-pan five or six minutes only. The mince when ready should resemble smooth putty, and should never present that hard granular condition so often seen in minces made from fresh meat cooked in the ordinary way. Beef mince should be served in a hot bowl and eaten with a teaspoon. Pepper and salt are the condiments recommended and a thin slice of toast, or toasted biscuit is usually allowed with each meal. It is not by any means easy in hot weather to keep the presses and mincers used in the preparation of beef juice and beef mince clean, but this is a matter which must always be strictly attended to. They should be taken to pieces as far as possible after use, and boiled in water to which carbonate of soda has been added.

In the treatment of bowel affections by medicine given by the mouth, the only drug which calls for special notice so far as the nurse is concerned, is Ipecacuanha as prescribed in dysentery. Ipecacuanha is usually given in powder, and in a single dose of from twenty to thirty grains, conveniently swallowed in cachets. Ipecacuanha should be given when the stomach is empty, and the patient be kept quiet and in the horizontal position for some hours after its administration. To prevent vomiting a sinapism is sometimes applied to the epigastrium, and the dose is guarded by opium, fifteen or twenty drops of the tincture, given about half an hour before the powder. Even when all precautions are taken, vomiting is often sudden and uncontrollable, and the nurse should make what preparations she thinks necessary to meet this, but should do so unobtrusively, to avoid as far as possible suggesting its occurrence to the patient.

Among cases of chronic diarrhœa there are always a few, distinguished as cases of sprue or psilosis, in which, owing to the state of the mouth, food of almost every kind is taken with difficulty. In these cases the mucous membrane lining the mouth and covering the tongue suffers from a more or less extensive loss of its protecting epithelium, and this is associated from time to time with the appearance of aphthous-looking spots and the formation of superficial ulcers. In a well-marked case a condition may in the end be established in which mere contact with food causes suffering, and all the movements connected with swallowing and mastication become difficult and painful. In feeding such patients the nurse will have to exercise a good deal of both patience and perseverance, for it is essential that a sufficiency of nourishment be taken however it is administered. Milk, a bland fluid which has the advantage over all other forms of nourishment in containing without any addition all that is absolutely needed for the support of the body, will usually be found the best food to use, and it certainly gives the least trouble to the patient. Concentration by evaporation has one of its best applications in cases of the kind, where it is desirable to shorten the period of administration and at the same time secure that a sufficient quantity is taken. Milk should be given lukewarm; extreme degrees of either heat or cold are always objected to by patients suffering from sprue. When beef juice or beef mince is ordered condiments will have to be avoided as far as possible. Jellies, as milk and calves' feet jellies, are easily swallowed, and the nurse, under the direction of the doctor, may have an opportunity given her for the exercise of her ingenuity in the preparation of these so that they may be at the same time nutritious and palatable.

In bowel cases long under treatment the patients should be frequently weighed, and a record kept of the results: when this is entrusted to the nurse she should be careful to see that the weights are taken at the same hour of the day—preferably before breakfast—and that due allowance is made for any change in the clothing of the patient.

*Fevers.*—Volumes have been written on the varieties, course, and treatment, of fevers in the tropics, but in all this the nurse is only indirectly concerned; her duties are little affected by varieties in type; and the nurse, who is qualified to undertake the case of enteric fever in England, has very little to learn regarding the management of similar cases abroad, however widely these may differ in the points which distinguish them as belonging to a particular class, or as due to a special cause.

In the treatment of fevers in the East quinine is a drug so frequently prescribed that a few lines may be usefully devoted to the subject of its administration. In malarial fever the large dose ordered is generally directed to be given in the sweating stage, when the temperature has fallen. When it is possible to choose the time of administration it is better to give these large doses at night before the patient goes to sleep, and in this way to lessen the discomfort of the cinchonism they may occasion; if given in the morning it should be after breakfast, when the stomach contains food. It is often difficult to get children to swallow a sufficient dose of quinine; on the whole, the drug is best given to children in sweetened milk; and if the child can be induced to eat a piece of well-buttered bread—not a difficult matter if sugar is added to the butter—before taking the medicine, the bitter taste and its persistence will certainly be lessened. The administration of a large dose of quinine to a woman who is pregnant is regarded by many as attended with risk, and it is the practice of some physicians, when obliged to prescribe this, to guard the dose with chlorodyne or some preparation of opium. Quinine may be given by the bowel in a suppository or enema, and by the skin as a subcutaneous injection; but the best method of administration in any particular case is of course a matter for the doctor to decide. Tabloids of quinine are convenient, but are apt to harden if kept for any length of time; when this is the case they should be crushed before being taken.

(*To be continued.*)

### Echoes from the Outside World.

AN OPEN LETTER TO A HOSPITAL NURSE.

General Buller has come home, and it has been proved that those who feared he might not be welcomed as he deserved, did not understand the British public. The latter may growl and snarl sometimes when things go wrong; but once let a man show dogged perseverance and courage under difficulties, and the "B.P." forgets the disaster and remembers the triumph. Southampton seems to have enjoyed itself vastly, and gave the hero the splendid greeting which was his due. Sir Redvers seemed to enjoy it, too, in his quiet way, more especially when he could talk about his men. Two facts which he brought to light about the difficulties of our Army are new and striking. One is that our men being mostly city born, and unaccustomed to using long sight, were at an immense disadvantage in the open country where the vision of the enemy extended at least two miles further than the average sight of the Englishman. Also, he pointed out, that the Boers could gather information at every native kraal, because they knew the language, whereas we had to get our news through interpreters—a very different thing.

According to a private letter received from Hong Kong, there is still a good deal of anxiety in the colony. My correspondent has lived there fifteen years, and you may remember I wrote about her at the time of the Peking trouble stating that she always kept a loaded revolver in her wardrobe. She alludes to this fact again this mail. "So far I have not had to make use of the revolver, and don't imagine I shall. Still, it is safer, and all the servants know I have it; they know also that I should not be backward in using it, and that I never give in to them. They always talk outside a good deal amongst themselves, and are well aware who fears them and who does not. I have a most insolent and bad-tempered washerman whom I keep on the premises. I can't stand the clothes going down to be washed in a plague-stricken quarter, or in a stream which runs over a plague cemetery. He refused to obey my orders, and shook his fist in my face. So I said, 'I will teach you who is master here,' and I sent up to the police station, which is just behind us, and got the inspector down. John Chinaman became humble at once. But the next week he disobeyed my orders again, and because I said he *should* do as I told him he ran off and left all the clothes dirty. I put on my hat and went after him, and threatened that if he did not come back and finish at once I should take him into the police court. He returned and has been quite meek ever since. He is a splendid washerman, and that is why I did not let him go. We are all very concerned as to what the Home Government are going to do about affairs up North. I hope they won't climb down. The Chinese are already saying we are afraid and are running away. Hong Kong is full of soldiers, and I fear our water supply will get short unless we have more rain next month. A friend who has just come back from the front says that the looting was awful, and that it seemed to make the men perfectly wild. He gave up what he took personally, but he brought his wife home two coats, one lined with silver fox skin, which reaches right down to her heels. He saw men going about with bars of silver and gold, laden with most costly furs, and some even had long strings of diamonds, and rows of brooches, watches, and lockets hung round their necks." The letter is very interesting, but it helps to explain why the Chinese people hate foreigners.

You probably read the annotation in the Hospital of November 3rd on "Filth and Fashion," in which the subject of trailing skirts in the public streets was treated from a medical, as well as a commonsense, point of view. This was succeeded by a letter in the *Times* on the same subject, and has led to the proposed formation of an Anti-Trailing League. A German Princess has been so much impressed with the evils which follow in the train of so foolish a custom —I did not intend a pun—that she has intimated her desire to give such a league every support, and also to do all she can towards forming one in her native country. Should a definite society for the suppression of trailing skirts be started in England, there is not the slightest doubt that it would soon number hundreds of members, but I am afraid that the offending class would be the most difficult to reach. The Princess of Wales and her daughters, it is well known, though, wearing long skirts for dressy, indoor wear, always don most sensibly-short gowns for walking along the Sandringham lanes; and it is of small moment whether wealthy women of fashion wear long skirts or short ones, because, with a few wise exceptions, all the exercise they take is what is strangely called "carriage exercise." But those who wear the longest trains, and are the best road sweepers are the women who, though unable to keep carriages, still wish to appear to belong to the upper ten, and hope, by strict attention, to every whim of Dame Fashion, to make people believe that they do. This class would be afraid to cut off their trains lest they should cut off the delusion at the same time. Still, there is one point which naturally suggests itself. If women in the highest circles join the league, and cause the fact to be widely circulated, the lower strata of society are sure to follow suit sooner or later. For the sake of health and cleanliness that day will indeed be welcome.

To those of us who laboured under the delusion that, at this latter end of the Nineteenth Century, birching was only allowed at boys' public schools or in prisons for criminal offences, Miss Honnor Morten's statements with regard to some of the girls' industrial schools have come with a rude shock. It appears that, according to Miss Morten's assertions, a girl of fifteen who had been guilty of disobedience received six "slaps" with a birch, and had her mouth tied up. Another, a few years younger, was sentenced to twelve strokes with a leather tawse, and was labelled "thief" for stealing food. These particular incidents came within Miss Morten's own knowledge, but it is most probable that they are by no means unique. It would seem, however, that the fault does not lie so much with the industrial schools themselves, as with the establishments in which the children have to be "farmed" out, owing to the incapacity of the Board to accommodate more than 900 children out of the 4,000 of whom it has to take charge. Still the Board are in reality the offenders for allowing those under their care to go anywhere except into such homes as they know are properly managed, and where, if punishment has to be given—and with the class of children usually committed to an industrial school it is, of course, impossible to be avoided —it is of a less primitive type than that which at present seems in vogue. The suggestion of Mr. Macnamara, at the same meeting, gave further food for reflection of a different sort. He said he thought "it would be a great thing for London if they could take 20,000 of the most wretched children out of the control of their parents," and place them in industrial schools. How dreadful it seems that there should be, roughly speaking, some 8,000 or 10,000 parents in this great metropolis quite unable, if not unwilling, to look after their offspring, and that in consequence the better classes should be obliged not only to clothe, educate, and look after their own children, but also to pay for the entire maintenance of those whose proper supporters are their own parents.

Those who were fortunate enough to get into Queen's Hall on Saturday afternoon—a great number who intended to attend the concert never got beyond the door, so great was the crush—had a treat indeed. M. Ysaye was at his very best. Even those who make a point of never missing a single concert at which he performs confessed that his rendering of the Beethoven concerto was, if poisible, grander than ever, and no wonder we all went mad for a few moments, and applauded with all the enthusiasm of which we were capable. The orchestra, too, was splendid in Schubert's "Unfinished Symphony," and the Egmont Overture. Miss Amy Sherwin's two songs by Mozart and Deliber were sung in her usual refined and pleasant style, and altogether it was a most enjoyable afternoon.

### The Mafeking Sisters of Mercy.

BY A CORRESPONDENT.

"Another interview!" sighed the Sister who kindly saw me at the convent in Crispin Street on Wednesday, and she told me that the Mafeking Sisters, although belonging to the same order as the Sisters there, had only been staying in Crispin Street. "They would be correctly described as the Sisters of Mercy from Mafeking," she said; "theirs is quite a separate house from ours; they are Irish, and, like other Sisters, had a little training in nursing during their novitiate, which enabled them to take up the work when it became necessary. They really belong to a teaching order."

"And they were presented at Windsor yesterday? What did they think of the interview?"

"The Queen was most kind and gracious; she thanked them for what they had done, and asked them many questions about the war."

"Did they receive any decoration?" I asked.

"No, I do not think anyone will until the campaign is over."

Notwithstanding the Sisters' natural dislike to the publicity which has been accorded them during the last few days in consequence of the Royal honour they have received, the Crispin Street Sister is of opinion that good work, though it may be nothing very extraordinary, is always an example to others, and should be rightly appraised.

"They dislike attracting so much attention," she said, "but for my own part I am proud of them."

### Everybody's Opinion.

THE EYE, EAR, AND THROAT HOSPITAL, CHELTENHAM.

THE MATRON OF THIS HOSPITAL writes: Having seen in last week's NURSING MIRROR that the above hospital had three beds, I beg to state that for two years now we have had *ten*.

"KILBURN SISTERS."

"A SISTER OF THE COMMUNITY OF ST. PETER" writes, at the request of the Rev. Mother, St. Peter's Home, Mortimer Road, N.W.: In last week's HOSPITAL there is a paragraph saying that the *Kilburn Sisters* have sent nurses from Korea to Wei-hei-Wei. May I ask you to alter this in your next issue, contradicting the term "Kilburn Sisters"? It is the Sisters of the Community of St. Peter, Mortimer Road, N.W., who sent the nurses from Korea, and we have no connection with the Kilburn Sisters (or Sisters of the Church Extension). As we are frequently confused with them we shall be much obliged if you will kindly rectify this error.

[We do not see that we were guilty of any error. There is a sisterhood at 27 Kilburn Park Road, and there is another at Mortimer Road, and colloquially the members of each would appear to have equal rights to the title of "Kilburn Sisters." It appears, however, that the St. Peter's Sisterhood disclaim the title.—ED. THE HOSPITAL.]

VISITING NURSES TO THE MIDDLE CLASSES.

"ANOTHER OLD ST. THOMAS'S NURSE" writes: "I would just like to add a word on the nurse's side as regards *her* comfort. Of course, it is *most* desirable that she should have every opportunity for rest on her return from either visiting or district work, and that the 'kind landlady with the fire and hot meal should be at hand.' It is always possible in a large town, or in an agricultural district, to find comfort and cleanliness in lodgings. Living in a mining district I have found *cleanliness* always wanting, and have had to take a house to ensure that my life shall be bearable and that I may not find in my own home what is too often so objectionable in the patient's. I wait entirely on myself, except perhaps a weekly cleaning. Oil stoves and wood fires lighten labour, dinners and suppers out also help. The work is congenial and the love for it is great. It would be a great sacrifice to me to relinquish it. There are many in the same position. What, I ask, can we do?"

THE CASE OF NURSE DOWLING.

MISS AGNES DOWLING writes: "I shall be much obliged for the favour of a little space in your columns. I write not merely on my own behalf, but on behalf of my fellow-nurses and fellow-workers in general. We are all at the mercy of those who employ us. I have been a nurse since '92; I have had experience of hospital work in three hospitals; I have been employed by three private nursing homes; and I have arrived at the conclusion that vast reforms are wanted in the system of treatment of nurses both in and out of hospital. As I have faced the lunatic asylum and stood in the witness-box in support of my principles, I think at least that I deserve a hearing. Both in hospital and out of it—that is, in nursing homes and private homes—nurses are overworked, underfed, underpaid, bullied, slave-driven, and made to feel constantly that life is a burden. Hospital life to the poor probationer is a veritable tread-mill, and the life in private nursing homes may be compared in many respects to life in a prison. Matrons sit in easy chairs and read novels, and every nurse on the staffs they employ is made to do the work of three women. There is no pity for a nurse when she is ill; no welcome for her when she returns sad and weary from death-bed scenes; no mercy for her if she protests, as I did, against overwork and harsh and unjust treatment. Matrons and doctors can do what they like where nurses are concerned. After two years steady work, I was refused a testimonial, because it was the matron's principle not to write testimonials; and because I threatened to show up the system in a home where I was barbarously treated I am now libelled permanently as insane, and deprived of the means of earning my living. With regard to my lawsuit against Dr. Dods, I think if it concerned someone else and not me the points about it which would most strike me would be the action of the jury who gave me a verdict and damages, in siding with the weaker side, and the unanimity of four judges in siding with the defendant and his supporters. I sat one day in the Court of the late Lord Chief Justice and someone beside me, looking at the walls, said: "This is a precious dirty place." I said, "Yes, in every sense of the word"—I meant in a moral as well as in a material sense. It is exemplified in the Royal Courts of Justice every day that there is one law for the rich and another for the poor. As the lunacy laws now stand, any doctor may write a libellous certificate of lunacy about anybody, and then screen himself behind a plea of privilege. The judges who administer the laws, the medical profession in general, and the medical press will, of course, champion his cause. I applied to several patients and friends of patients to help me in my time of trouble. Nearly all refused. One lady whose father I nursed, very nearly assaulted me for asking her to come to the trial as a witness. She refused to come, and sent me three solicitors' letters. My advice to intending probationers is: "Give the idea up. Keep out of hospital, and keep clear of matrons."

### Presentations.

LADY HOZIER CONVALESCENT HOME.—Miss Gordon, late matron of the Lady Hozier Convalescent Home, Lanark, has been presented, on her retirement, with a very handsome gold bracelet from the managers of the institution.

WESTERN INFIRMARY, GLASGOW.—Last week a pleasant evening was spent by the nurses of the Western Infirmary, Glasgow, in the drawing-room of the Nurses' Home, the occasion being a presentation to Miss More, assistant matron, in connection with her departure. At the beginning of the evening Dr. Mackintosh, superintendent of the institution, in a short congratulatory speech presented Miss More, on behalf of the nursing staff with a handsome silver salver, silver tea set, and half a dozen silver tea spoons. Miss More had previously received the gifts of a silver-backed hand mirror and silver-topped perfume bottle from the maids connected with the hospital and home.

### For Reading to the Sick.

MY strength is made perfect in weakness.—2 *Cor.* xii. 9.

I could not do without Thee,
I cannot stand alone,
I have no strength or goodness,
No wisdom of my own;
But Thou, beloved Saviour
Art all in all to me,
And weakness will be power
If leaning hard on Thee.
186, *Hymns A. and M.*

Leaning on Thee, my Guide, my Friend,
My Gracious Saviour! I am blest;
Though weary, Thou dost condescend
To be my rest.

Leaning on Thee, this darkened room
Is cheered by a celestial ray;
Thy pitying smile dispels the gloom,
Turns night to day.

Leaning on Thee, I breathe no moan,
Though faint with languor, parched with heat,
Thy will has now become my own;
That will is sweet.

Leaning on Thee, with child-like faith,
To Thee the future I confide;
Each step of life's untrodden path
Thy love will guide.—*C. Elliott.*

Many say sadly, when they are called aside for awhile to rest, "I have no more opportunities now of doing good."

God keeps no cumberers upon His ground; if thou wert indeed of no use, child of God, surely thou wert not here.

Now is the time for Prayer; prayer for those around thee, prayer for the place where thou livest, for thy ministers, thy friends, thine enemies, any cause thou hast at heart.

Thine opportunity is for Prayer, use it to thine ability, and be content.

Then, too, thy patience and thy cheerful resignation now can teach those who see thee how the grace of God can triumph over every affliction.

Thou art thyself the means of drawing out the charity of those around thee; let that again comfort thee.

Thy weakness can draw forth their strength, thy helplessness their tenderest unselfishness, thine infirmities the exercise of their best gifts of love and pity. Is it nothing to do this?

Surely we may rejoice that God employeth us as His instruments, even at such a cost to ourselves.

Thy sufferings are not in vain; none ever were that were quietly, patiently and serenely borne for Christ's sake; thou hast thy niche to fill, thy little light to shed; thy slender thread of life is needed still in the great web of God's eternal plan; and darker shades bring out the glories of the brighter spots, and thou art necessary, even thou, unto the Master Weaver's work of Love.—*E. A. D.*

Bear on—for He hath loved thee, and thy love
Shall change to gems and glitter on thy brow
In the amber light of the eternal years;
And thy worn soul, so chafed and fretted now
Shall know the freedom He has won for thee
In the love shadows of Gethsemane.—*S. Doudney.*

### Novelties for Nurses.

### SANITARY HOSEZENE TOWELS.

WE desire to draw the attention of our readers to the comfort and convenience of this toilet requisite for ladies. For travelling and other occasions where laundry work is not always procurable we can imagine no greater convenience, as the towel can be burnt after use. It possesses the further merit of being hygienic, and is also soft, light, and absorbent. They are made in one quality only, the price varying with the size. The Hosezene Company, Nottingham, will have pleasure in forwarding a sample packet to any address on receipt of application, and we feel sure that a trial will result in further orders.

### Notes and Queries.

**The Editor is always willing to answer in this column, without any fee, all reasonable questions, as soon as possible.**

**But the following rules must be carefully observed :—**

1. **Every communication must be accompanied by the name and address of the writer.**
2. **The question must always bear upon nursing, directly or indirectly.**

**If an answer is required by letter a fee of half-a-crown must be enclosed with the note containing the inquiry.**

*Maternity Training.*

(69) Which is the best school for training midwives and monthly nurses? Can any preparation be made prior to entering for a course of training?—*S. T. N.*

See the "Nursing Profession, How and Where to Train" for full particulars as to the various institutions for training maternity nurses; and write to the Secretary, The Midwives' Institute, 12 Buckingham Street, W., for information as regards preliminary studies.

*Children's Nurses.*

(70) Can you kindly give me the address of any institution where women can be trained as children's nurses only?—*R. C.*

The Norland Institute for Training Children's Nurses, 29 Holland Park Avenue, Notting Hill, London, W., and the Liverpool Ladies' Sanitary Association, 8 Sandon Terrace, Liverpool.

*Male Nurse.*

(71) I should esteem it a favour if you would kindly tell me how and where I can get training as a male nurse.—*A. S. E.*

The National Hospital for the Paralysed and Epileptic, Queen's Square, Bloomsbury, W.C., and through any of the asylums training its attendants for the Medico-Psychological Societies' Certificates. For lists of asylums see the "Nursing Profession, How and Where to Train" (2s., Scientific Press).

*Register.*

(72) Do you keep a register of doctors and nurses who receive private patients? I see in your queries that someone wishes to find a home for the Weir Mitchell treatment for nerve depression, and as I am quite used to nursing that complaint, I thought you might help me. I am enclosing stamped envelope for reply.—*Marion R.*

We should be very pleased to help you, and have forwarded your letter to our correspondent. But we keep no register, and 2s. 6d. must accompany letters needing a private reply.

*Visiting Nurse.*

(73) I am thinking of starting visiting nursing. Would you kindly give me list of fees? I see somewhere in THE HOSPITAL that the charge is 3s. 6d. per hour, but that is more than my proposed class of patients could afford.—*A. T.*

See reply to "Quietness." Fees must always depend largely if not chiefly upon the distance to be travelled between case and case. If a nurse could make a living out of a couple of streets much lower fees than the above would be reasonable recompense.

*Supplementary Certificate.*

(74) A nurse with two years' training in a general hospital and three years' experience in private work is anxious to get a three-year certificate. Is there any institution where she could train over again without a premium, either for three years or for one, in order to obtain it?—*Anxious.*

Write to the Matron, the Victoria Park Hospital, E. She offers two years' supplementary training to nurses with good credentials for previous general training, and obtains for them a three years' certificate.

*Male Nurse.*

(75) Can you kindly tell me of a hospital or asylum where a young man of 20 could be trained as nurse, and also say what salary he would receive?—*Anxious.*

See reply to "A. S. E." At the National Hospital for the Paralysed and Epileptic the salaries of the male nurses begin at £12 and rise to £20 the second year. The lowest age at which candidates are admitted is 21 years.

*Incurable.*

(76) Could you tell me of a permanent home in or near London if possible where a lady unable to walk and in bad health would be admitted at the low terms of 12s. 6d. a week?—*F. M. R.*

The Woodside Home, Whetstone, N., is a permanent home for incurable gentlewomen; also the Helena Nursing Home, Brownlow Road, Reading.

*South Africa.*

(77) Is there any institute in South Africa which engages nurses with three years' certificate for fever nursing, and the L.O.S.? Where would be the best place to train for the L.O.S. certificate?—*H. T.*

The Victoria Nurses' Institute, Cape Town, Cape Colony (write first for particulars as to engagement). Any of the lying-in hospitals would train you well. Queen Charlotte's is the most important.

*Nurse-child.*

(78) Will you kindly tell me how I can get a nurse-child for a well-recommended, kind motherly woman to rear (not adopt), minimum charge 6s. a week?—*Nurse L. H. M.*

Write to Miss Mason, Senior Inspector of Boarding-out under the Local Government Board. Address, Local Government Board, Whitehall, S.W.

### Standard Books of Reference.

"The Nursing Profession: How and Where to Train." 2s. net; post free, 2s. 4d.
"The Nurses' Dictionary of Medical Terms." 2s.
"Burdett's Series of Nursing Text-Books." 1s. each.
"A Handbook for Nurses." (Illustrated.) 5s.
"Nursing: Its Theory and Practice." New Edition. 3s. 6d.
"Helps in Sickness and to Health." Fifteenth Thousand. 5s.
"The Physiological Feeding of Infants." 1s.
"The Physiological Nursery Chart." 1s.; post free, 1s. 3d.
All these are published by THE SCIENTIFIC PRESS, Ltd., and may be obtained through any bookseller or direct from the publishers, 28 and 29 Southampton Street, London, W.C.

### Travel Notes.

By Our Travelling Correspondent.

### LXI.—ON WINTERING ABROAD.

Nothing is more difficult to decide by the inexperienced, than what they can by the exercise of strength of mind, finally bring themselves to leave behind. The more I travel, the less I take, and yet even now I often find I might with great advantage have done without several articles that cumber my trunk. Amongst these annoying extras, I should place first and foremost a superfluity of clothes.

### What to Take and What to Leave Behind.

The greatest heart searchings are felt over evening dress. Now believe me you want but little of this. You are not at all likely to mix in smart society, and if you do go out to a few evening parties (extremely unlikely), a black silk skirt with a pretty blouse, preferably of some thin material of the same hue, will meet all your requirements, and the black silk skirt with morning blouses can be utilised in warm weather.

Above all things let me caution you to have sufficiently warm clothes; it is a rooted belief that the Riviera, Florence, or Rome, are always warm. Nothing can be a greater mistake; all these charming places can be extremely cold. The great difference to our own climate lies in the fact of their being usually flooded with sunshine and free from damp. I consider that you need clothes quite as warm as in England, and recommend nothing but woollen of varying degrees of thickness, both for outside and inside wear. Never use cotton or thread stockings, but always woollen or silk, the stone floors of the Italian churches can be cruelly cold. The genial warmth of the winter sun in Italy presents a serious danger, but one easily guarded against. Always carry a light wrap to throw over your shoulders in passing from sunny to shady streets, and especially when you enter the churches.

I consider three dresses sufficient and four a handsome allowance for a six months' residence abroad, but remember that they should all be in good condition, no wearing out of old clothes, a most pernicious habit belonging to the travelling British female, and one that brings its own punishment, for after a month or six weeks' hard service, these aged garments begin to give out treacherously and one reaps one's reward in burst seams, thin places, and a general feebleness of constitution developed in the hems of dresses. I once paid nearly 8 francs for a new skirt braid and a modicum of renewed lining to the bottom of the garment. It taught me a lesson.

Four good pairs of stockings, also new, will be required; and be very careful in the selection of your footgear. For six months you will require one fairly stout pair of boots for wet weather and country walking, and two pairs of Oxford shoes. One of these latter should be tan—they are much cooler and also easier to clean. One pair of indoor shoes and another of a smarter kind, if you intend evening gaieties, sufficient.

### The Question of Wraps.

Do not stint yourself in these necessary articles. First of all a light mackintosh; secondly a substantial and really warm cloak of the golf pattern; and thirdly, a much lighter cape also in that form that can be carried over the arm without fatigue, and put on at the slightest feeling of chilliness. I think a dust cloak of alpaca is a great convenience, and is no weight in the luggage. White dust soon makes garments look shabby and feel dirty. Always take with you a shawl of good wool, it is more useful to supplement insufficient bedclothes than the heavier railway rug.

### Household Conveniences.

It is difficult to advise on this knotty point, because so much depends on whether you are rich or poor, whether you are strong, or more or less of an invalid, and whether you mean to live in an hotel or in apartments, or more humbly, in a room or rooms and "do" for yourself. This last arrangement can only be carried out by those who speak the language of the country fluently, and do not mind decidedly roughing it. I have often done it myself, but then I am of an adventurous disposition and I freely confess that my arrangements sometimes left something to be desired.

We will, then, consider that you are to live in an hotel or pension, opulent or humble according to your means. First of all take tea with you, one pound is allowed by the authorities if you declare it as for your "propre service." It is very dear in France or Italy, indeed anywhere on the continent, and not usually good, because it is not kept hermetically sealed.

A tea basket is almost an essential of existence to the travelling English woman; they are expensive things to buy, but mine arranged for two people cost me only 3s. 4d. if I remember rightly. I need hardly say it was a home-made article, but has proved itself of surpassing value, and has journeyed with me many thousands of miles.

Any one wishing to construct for themselves a similar treasure shall receive full instructions. Table and bed linen are always plentiful abroad, but take two or three tea-cloths with you for washing up your tea things and many other purposes, two packets of Hudson's washing powder will be useful for scalding teapots and milk-jugs, &c. . . . and for washing your own stockings; foreign laundresses develop such a remarkable aptitude for losing the half of a pair of stockings that I never now trust mine in their clutch. A small common tin kettle value 10d. to really go on the wood fire will be found a luxury. Kettles except enormous family receptacles are unknown. You should take with you a very small bottle of brandy for the journey, and buy if you want more; it is good and not dear on the Continent. Take also a little castor oil, some chlorate of potash for a sore throat, and a little oil silk. Ink is dear and bad; besides your travelling ink-bottle *full*, take another penny bottle; you will probably not want more. An indiarubber bath is an immense convenience; they are to be had large enough for all but those of an "out" size for 10s. 6d., and they fold up delightfully into a small bag, occupying no more room than a bath sponge. Another item of which to take a good store is tooth-powder. Chemists' wares are very dear; tooth-powder and soap (the latter very nasty) cost a great deal, and should always be bought in England. I find I have left myself no room to speak of sketching materials, and I must leave that subject for another day, as well as the important question of trunks and boxes.

### TRAVEL NOTES AND QUERIES.

Rules in Regard to Correspondence for this Section.—All questioners must use a pseudonym for publication, but the communication must also bear the writer's own name and address as well, which will be regarded as confidential. All such communications to be addressed "Travel Editor, 'Nursing Mirror,' 28 Southampton Street, Strand." No charge will be made for inserting and answering questions in the inquiry column, and all will be answered in rotation as space permits. If an answer by letter is required, a stamped and addressed envelope must be enclosed, together with 2s. 6d., which fee will be devoted to the objects of the "'Hospital' Convalescent Fund." Any inquiries reaching the office after Monday cannot be answered in "The Mirror" of the current week."

Rome (Pilgrim).—The water is excellent, and to be taken without fear however, no one can answer for all the receptacles it may pass through, therefore, to make all safe, boil all you drink yourself. The English congregate mostly about the Piazza di Spagna, and apartments there are expensive in consequence. If you think seriously later on of going, I can tell you of several Pensions where your terms, or something like them, will be accepted; for a short stay such as you propose I think apartments would be a mistake.

Innsbruck for Winter (Penelope).—It is a delightful place for those who are fairly strong, can get about, and do a few mild excursions. For a semi-invalid, I do not consider it quite so suitable, because there are not so many places of interest in the way of art galleries and churches to be studied in inclement weather. Rome and Florence are rich in these respects, and are, therefore, excellent for those who cannot make much exertion.

